# Generation and Characterization of a Virulent *Leptosphaeria maculans* Isolate Carrying a Mutated *AvrLm7* Gene Using the CRISPR/Cas9 System

**DOI:** 10.3389/fmicb.2020.01969

**Published:** 2020-08-11

**Authors:** Zhongwei Zou, Fei Liu, Carrie Selin, W. G. Dilantha Fernando

**Affiliations:** Department of Plant Science, University of Manitoba, Winnipeg, MB, Canada

**Keywords:** *Leptosphaeria maculans*, canola, gene editing, breeding for disease resistance, *Rlm7*, *AvrLm7*, CRISPR/Cas9, *Brassica napus*

## Abstract

Blackleg, caused by the fungal pathogen *Leptosphaeria maculans*, is the most important disease affecting canola (*Brassica napus*) crops worldwide. We employed the clustered regularly interspaced short palindromic repeat (CRISPR)/CRISPR-associated (Cas) system to generate the mutant isolate umavr7 from a point mutation of the *AvrLm7* coding region in a *L. maculans* isolate (UMAvr7). Reverse transcription PCR and transcriptome data confirmed that the *AvrLm7* gene was knocked out in the mutant isolate. Pathogenicity tests indicated that umavr7 can cause large lesions on a set of *Brassica* differential genotypes that express different resistance (*R*) genes. Comparative pathogenicity tests between UMAvr7 (wild type) and umavr7 on the corresponding *B. napus* genotype 01-23-2-1 (with *Rlm7*) showed that umavr7 is a mutant isolate, producing large gray/green lesions on cotyledons. The pathogenicity of the mutant isolate was shifted from avirulent to virulent on the *B. napus Rlm7* genotype. Therefore, this mutant is virulence on the identified resistant genes to blackleg disease in *B. napus* genotypes. Superoxide accumulated differently in cotyledons in response to infection with UMAvr7 and umavr7, especially in resistant *B. napus* genotype 01-23-2-1. Resistance/susceptibility was further evaluated on 123 *B. napus* genotypes with the mutant isolate, umavr7. Only 6 of the 123 genotypes showed resistance to umavr7. The identification of these six resistant *B. napus* genotypes will lead to further studies on the development of blackleg disease resistance through breeding and the identification of novel *R* genes.

## Introduction

Blackleg, caused by the fungal pathogen *Leptosphaeria maculans*, is the most important disease affecting canola (*Brassica napus*) crops worldwide (Fitt et al., 2006). Since the 1990’s, blackleg has been identified as a major disease in Canadian canola and has resulted in significant yield losses (Gugel and Petrie, 1992). Blackleg disease severity and the extent of canola yield loss vary with geographic distribution, climate, cultivar and crop rotation. In the last four decades, blackleg has been mainly controlled by the use of resistant cultivars and crop rotation (Fernando et al., 2016). However, *L. maculans* is considered a highly virulent and globally invasive species (Fitt et al., 2006; Zhang and Fernando, 2018). Previous studies have indicated that a gene-for-gene relationship exists between each *R* gene in canola and its corresponding avirulence gene (*Avr*) in the *L. maculans* isolate (Ansan-Melayah et al., 1998; Balesdent et al., 2002). *R* gene-mediated resistance, based on this relationship, is used so frequently that many blackleg disease management strategies have been developed, and cultivars have been bred for resistance to the fungal populations that harbor the majority of the corresponding avirulence alleles (Daverdin et al., 2012).

To date, a total of 16 *Avr* genes have been identified in *L. maculans*, including *AvrLm1-11*, *AvrLepR1-LepR3*, and *AvrLmS* (Gout et al., 2006; Hayward et al., 2012; Van de Wouw et al., 2014; Ghanbarnia et al., 2015; Plissonneau et al., 2016). Among these, nine avirulence genes have been cloned: *AvrLm1* (Gout et al., 2006), *AvrLm3* (Plissonneau et al., 2016), *AvrLm6* (Fudal et al., 2007), *AvrLm5(J1)* (Van de Wouw et al., 2014), *AvrLm2* (Ghanbarnia et al., 2015), *AvrLm4-7* (Parlange et al., 2009), *AvrLm11* (Balesdent et al., 2013), *AvrLm9* (co-segregating with *AvrLm5*) (Ghanbarnia et al., 2018), and *AvrLm10* (Petit-Houdenot et al., 2019). A recent study indicated that *AvrLmJ1* maps to the same position as *AvrLm5*; isolates carrying the *AvrLmJ1* locus confer avirulence toward *Brassica juncea* genotypes with the *Rlm5* gene, demonstrating that *AvrLmJ1* is *AvrLm5* (Plissonneau et al., 2018). Correspondingly, various major resistance genes in *Brassica* species have been identified, including *Rlm1-11*, *LepR1-LepR4*, *RlmS*, *BLMR1*, and *BLMR2* (Chèvre et al., 1997; Rimmer, 2006; Van de Wouw et al., 2009; Eber et al., 2011; Long et al., 2011). Among these, *Rlm5* and *Rlm6* are found in *Brassica juncea* (AABB genome) (Chèvre et al., 1997; Balesdent et al., 2002; Van de Wouw et al., 2014). One resistance gene, within two alleles of *LepR3*/*Rlm2*, which interacts with *AvrLm1* and *AvrLm2*, respectively, has been cloned (Larkan et al., 2013 and 2015).

In the past decade, the predominant tools employed to achieve site-directed double strand breaks are ZFNs (zinc-finger nucleases) and TALENS (transcription activator-like effector nucleases), both of which have provided invaluable assistance in targeted gene mutation in a diverse range of organisms. More recently, the clustered regularly interspaced short palindromic repeats (CRISPR)-Cas (CRISPR-associated) protein system has gained much attention and has been widely used for editing the genomes of animals, plants and fungi. In comparison to ZFNs and TALENS, CRISPR/Cas9 is easier to manipulate, as it employs a RNA-guide nuclease, Cas9, to induce double-strand breaks for mutation and can be recycled to edit different targets. CRISPR/Cas9 has been used for targeted mutagenesis in various plant species, including *Arabidopsis* (Jiang et al., 2013), *Nicotiana tabacum* (Li et al., 2013), potato (Shan et al., 2013), rice (Xie and Yang, 2013), tomato (Brooks et al., 2014), and soybean (Cai et al., 2015). The CRISPR system has also been used extensively in filamentous fungi to decode fungal pathogenesis (Deng et al., 2017), and Takayuki et al. (2015) developed a modified tailor-made CRISPR/Cas system to increase targeted gene replacement efficiency in the rice blast fungus. Comparative analyses of secreted proteins in plant pathogenic smut fungi and related basidiomycetes have been conducted using the CRISPR/Cas9 toolkit for simultaneous disruption of multiple genes (Schuster et al., 2018). Very recently, Idnurm et al. (2017) optimized a CRISPR/Cas9 system to disrupt the *hos1* gene, encoding a predicted osmosensing histidine kinase, in different *L. maculans* strains. The *L. maculans* strains with the mutated *hos1* gene showed reduced growth under high salt conditions but were still able to cause lesions on canola plants.

From a 6-year blackleg disease survey, examining the dynamic avirulence allele profiles of *L. maculans* isolates in Manitoba, Canada, it was evident that the fungal populations carry a diverse number of *Avr* genes. A total of 180 *Avr* gene races were identified from 964 isolates, with three major races observed: *AvrLm-2-4-5-6-7*, *AvrLm2-4-5-6-7-S*, and *AvrLm-1-4-5-6-7-11-(S)*. Very few isolates carried a single *Avr* gene conferring virulence to most commercial canola cultivars (Fernando et al., 2018). However, a *L. maculans* isolate (DS103) with few *Avr* genes, termed UMAvr7, was identified and carried *avrLm1-2-3-4-9-11-LepR1-LepR2-S-AvrLm5-6-7*, which is virulent on *B. napus* genotypes with different resistance genes (except *Rlm7*). This isolate was considered the ideal candidate, not only for elucidating the role of *Avr* effectors in pathogenicity, but also with which to examine the role of *Avr-R* interaction in the *B. napus* host defense. For breeding programs, we were expecting an ideal isolate that does not carry any of the identified *Avr* genes in *L. maculans*, and therefore, can be adopted for new resistance gene identification in *Brassica* genotypes. *Brassica* genotype showing incompatible reaction to this isolate should have a corresponding novel resistance *R* gene. *AvrLm4-7* gene has been cloned and determined by a single nucleotide mutation in coding region, where codon^358^ shifted from “C” to “G” conferring recognition to *Rlm7* and *Rlm4/7* in *B. napus*, respectively (Parlange et al., 2009). To knock out *AvrLm7* gene for resistance screening in *Brassica* germplasm, we generated a deletion in *AvrLm7* by employing CRISPR/Cas9. To validate this gene deletion, we analyzed the pathogenicity of both UMAvr7 (wild type) and the umavr7 (CRISPR mutant) on the corresponding *B. napus* cultivars 01-23-2-1 (with *Rlm7*) and Westar (no *R* gene), along with sequencing. The main objective of this study is to obtain a suitable isolate (mutant) altering the pathogenicity on *B. napus Rlm7* genotype. We also evaluated an additional 123 *B. napus* genotypes to putatively reveal any unknown *R* genes screened by this isolate.

## Materials and Methods

### *Leptosphaeria maculans* Isolate Preparation, DNA Extraction and PCR Assays

A *L. maculans* isolate (DS103) (hereafter named UMAvr7) collected in a previous study, and carrying the *AvrLm5-6-7* gene, was identified (Fernando et al., 2018). The isolate was grown on V8 agar juice medium amended with 0.0035% (w/v) streptomycin sulfate under continuous florescent light at room temperature for one week, before being subjected to a second round of single-pycnidiospore isolation to obtain a pure culture. The pycnidiospore and mycelia on the plate were flooded with 3 mL of sterile distilled water, collected and used for subsequent inoculum preparation and DNA extraction.

Genomic DNA was extracted from a mixture of pycnidiospore and mycelia suspensions of purified *L. maculans* isolates, using the modified CTAB method, as described by Liban et al. (2016). We used PCR to confirm the presence or absence of cloned avirulence genes including *AvrLm1* (Gout et al., 2006), *AvrLm2* (Ghanbarnia et al., 2015), *AvrLm4-7* (Parlange et al., 2009), *AvrLm5-9* (Van de Wouw et al., 2014; Ghanbarnia et al., 2018), *AvrLm6* (Fudal et al., 2007), *AvrLm11* (Balesdent et al., 2013), and *AvrLm3* (Plissonneau et al., 2016). The primers used are listed in Supplementary Table S1.

### Phenotypic Characterization of the *AvrLm4-7* Allele in the UMAvr7 *L. maculans* Isolate

Avirulence gene profiles of *L. maculans* isolates were determined by inoculating the isolates onto a set of *Brassica* differentials harboring differently related resistance (*R*) genes (Supplementary Table S2). Seeds of eleven *Brassica* differential genotypes were each sown into 96-cell flats filled with ProMix BX (Premier Tech, Rivière-du-Loup, Québec, Canada) and grown in a controlled growth chamber at 16/21°C (night/day) with a 16-h daily photoperiod.

The concentration of harvested spores was adjusted to 2 × 10^7^ spores/mL for the cotyledon inoculation test. Cotyledons from each seedling (7 days old) were punctured on four lobes and inoculated with a droplet of 10 μL inoculum, as previously described by Zhang et al. (2016). A minimum of six plants of each genotype were used for the inoculation test. Disease symptoms in infected cotyledons were evaluated at 14 dpi (day post-inoculation) using a rating scale of 0–9, based on lesion size, necrosis/chlorosis and the presence of pycnidia (Williams and Delwiche, 1979; Zhang et al., 2016).

### Generation of Mutant Isolate umavr7

**FIGURE 1:**
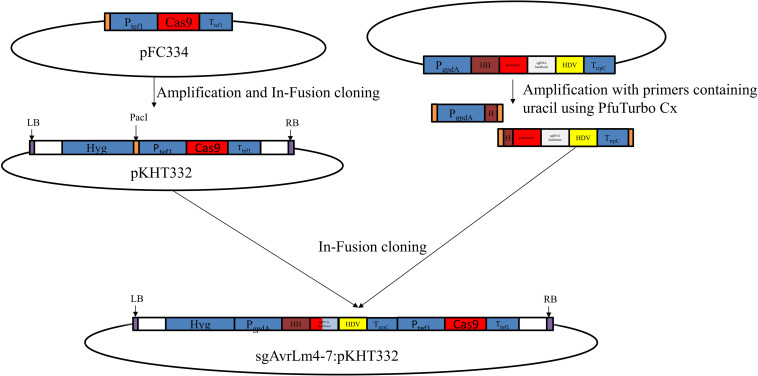
Diagrammatic overview of the establishment of CRISPR/Cas9 mediate system in *Leptosphaeria maculans AvrLm4-7* gene via in-fusion cloning strategy. Briefly, a fragment containing *Aspergillus nidulans tef1* promoter, *Cas9* gene, and *Aspergillus nidulans tef1* terminator was amplified from a pFC334 plasmid and then inserted into pKHT332 via In-Fusion cloning. *sgAvrLm4-7*:pKHT332 by inserting the *AvrLm4-7* sgRNA gene together with the gdpA promoter and trpC terminator into pKHT332 (restriction enzyme cutting by *Pac*I) by integrating two PCR fragments amplified from plasmid pFC334 via In-Fusion cloning.

To generate the vector pKHT332, used to deliver the CRISPR/Cas9 system into *L*. *maculans*, a fragment containing *Aspergillus nidulans tef1* promoter, *Cas9* gene and *Aspergillus nidulans tef1* terminator was amplified from a pFC334 plasmid (Nødvig et al., 2015) and then inserted into pKHT332 via In-Fusion cloning (Clontech, Takara Bio, United States) (Figure 1). The open reading frame of *AvrLm4-7* was submitted to the CRISPR Optional Target Finder^1^ to search sgRNA sequences. We selected sgRNA showing at least five mismatched base pairs with other non-targeted sequences as the candidate sequence to BLAST against the *L. maculans* genome. We generated *sgAvrLm4-7*:pKHT332 by inserting the *AvrLm4-7* sgRNA gene together with the gdpA promoter and trpC terminator into pKHT332 (restriction enzyme cutting by *Pac*I) by integrating two PCR fragments amplified from plasmid pFC334 via In-Fusion cloning (Takara Bio, Japan) (Figure 1), as described by Nødvig et al. (2015). PfuTurbo Cx Hotstart polymerase (Stratagen) was employed for amplification during In-Fusion cloning. Sequences of primers used for the constructs are shown in Supplementary Table S1. *Agrobacterium*-mediated transformation of UMAvr7 was conducted, as previously described by Gardiner and Howlett (2004). Briefly, the *Agrobacterium* with binary vector were cultured from a single colony and co-cultured with prepared *L. maculans* (DS103) spore solution filtered through sterile Miracloth and funnel. Then, the co-culture of *Agrobacterium* and *L. maculans* was poured to 25 mL IM plates (induction media) with AS (acetosyringone). After 48 h incubation at 22°C in the dark, the plates were overlaid with 25 mL 10% V8 medium with 100 μg/mL hygromycin. The plates were sealed and placed in a growth cabinet with lights at 22°C to enable *L. mauclans* mutant growth for 5–10 days. The generated mutants were grown on the V8 medium with hygromycin for positive colony selection. A single pycnidiospore was selected, cultured, and used for the following experiments.

### CRISPR/Cas9 Efficiency and Off-Targets Analysis

A total of 100 candidate isolates were generated and selected for pathogenicity testing to validate the actual strains harboring a potential deletion in *AvrLm7*. The cotyledon inoculation method was performed as described above. The isolates were maintained on V8 juice, and pycnidiospores were collected for genomic DNA extraction. To confirm the sequence variation in targeted sgRNA regions, candidate isolates were amplified and sequenced with Seq4-7 F/R primers (Supplementary Table S1). Sequence alignment was conducted by SeqMan in DNAstar^2^.

To analyze the potential off-targets of *sgRNAAvrLm4-7-Cas9*, the sgRNA sequence was employed to search the *L. maculans* genome database^3^. Sequence identity of at least 10 bp to sgRNA was selected to identify potential off-targets. Primers were designed for amplifying and sequencing the off-target regions in both wild type (UMAvr7) and mutant (umavr7) isolates (Supplementary Table S1).

### Reverse Transcription PCR for Expression Analysis of *AvrLm7*

The wild-type (UMAvr7) and mutant (umavr7) isolates were inoculated on cotyledons of 7-day-old seedlings of *B. napus* genotypes Westar (No *R* gene) and 01-23-2-1 (*Rlm7* gene). RNA was extracted from 100 mg of cotyledons sampled from 1 day and 11 days post inoculation using Plant RNA Reagent (Invitrogen, Carlsbad, CA, United States). The first strand cDNA was synthesized from one microgram of total RNA from each sample using RevertAid First Strand cDNA Synthesis Kit according to the manufacturer’s instructions (Thermo Scientific, Waltham, MA, United States). Reverse transcription PCR (RT-PCR) was performed with the primers designed from coding region of *AvrLm7* gene by 30 cycles of 94°C for 30 s, 58°C for 30 s, and 72°C for 30 s. Three biological replicates were sampled for RT-PCR analysis. *B. napus actin* gene was used as a control.

### Pathogenicity Evaluation of *L. maculans* Isolates UMAvr7 and umavr7

To compare the disease severity of the wild-type isolate UMAvr7 and its mutant (umavr7) generated from the CRISPR/Cas9 system, both isolates were inoculated onto punctured cotyledons of *B. napus* genotypes Westar and 01-23-2-1 seedlings 7 days after seeding, as described above. Lesion sizes were quantified at 14 dpi in Assess 2.0 (American Phytopathological Society, St. Paul, MN, United States). A total of six plants with 24 lesions were quantified and the experiments were repeated once. Analysis of variance (ANOVA) were performed using SAS Version 9.4 (SAS Institute, Inc., Cary, NC, United States).

### *In situ* Histochemical Detection of Hydrogen Peroxide

Hydrogen peroxide levels in the seedling cotyledons of *B. napus* cultivars Westar and 01-23-2-1 inoculated with wild-type and mutant isolates were detected via 3,3-diaminobenzidine (DAB) staining. The staining solution was prepared by dissolving 50 mg DAB in 50 mL sterile H_2_O (1 mg mL^–1^DAB solution), with the pH adjusted to 3.0 with 0.2 M HCl. After immersion in the DAB staining solution in the dark for 6 hours at room temperature, the stained cotyledons were then cleared using bleaching solution (ethanol: acetic acid: glycerol = 3: 1: 1). Hydrogen peroxide (H_2_O_2_) oxidizes DAB in the presence of peroxidases and produces a reddish brown precipitate (Daudi and O’Brien, 2012).

### Broadening the Genetic Resources of *L. maculans* Resistance in *Brassica napus* by Inoculation With Mutant Isolate umavr7

A collection of 123 *B. napus* genotypes, consisting of varieties and advanced breeding lines, were selected for the pathogenicity test using the umavr7 isolate under greenhouse conditions (Fernando et al., 2018). These *B. napus* genotypes included winter types and semi-winter types and were derived from the major canola growing areas of China. All *B. napus* genotypes were subjected to cotyledon inoculation tests with at least six plants of each genotype. The inoculum preparation, inoculation methods, rating, and resistance analysis used were described as above.

## Results

### Genotype Confirmation of the *L. maculans* UMAvr7 Isolate

A *L. maculans* isolate (DS103), termed UMAvr7, was originally identified in a previous study during a canola disease survey in Manitoba, Canada (Fernando et al., 2018). PCR analysis of this isolate, applied to determine the *Avr* gene profile, indicated that only the *AvrLm5*, *AvrLm6*, and *AvrLm4-7* genes could be positively amplified. Transcriptome analysis of inoculating this isolate on susceptible and resistant *B. napus* genotypes by dual-RNA sequencing also indicated that none of the *AvrLm1*, *2*, *3*, and *11* genes were expressed, except *AvrLm5*, *AvrLm6*, and *AvrLm4-7* genes (unpublished transcriptome data). Taken together, UMAvr7 is lacking most known avirulence genes that interact with the resistance genes from *B. napus* and was considered an ideal candidate for creating a more virulent *L. maculans* strain.

To confirm the PCR genotyping, UMAvr7 was inoculated on a set of *Brassica* differentials carrying the resistance genes *Rlm1*, *2*, *3*, *4*, 6, *7*, *9*, and *LepR1*, *R2*, *R3* to profile the avirulence genes. The results revealed that UMAvr7 caused disease symptoms on all tested *Brassica* differentials with the exception of the *B. napus* genotype 01-23-2-1 (*Rlm7*) and *B. juncea* genotype Forge (*Rlm6*) (Supplementary Figure S1). The disease rating scores ranged between 7 and 9 for the cotyledons of the differentials that displayed a susceptible reaction. As expected, *B. napus* genotype 01-23-2-1 (*Rlm7*) and *B. juncea* genotype Forge (*Rlm6*) displayed high levels of resistance (disease rating score of 1.23 and 2.67) to UMAvr7 (Supplementary Figure S1). Currently, we do not have a proper *B. juncea* genotype carrying *Rlm5* to characterize the phenotype consistent with the *AvrLm5* genotype in the isolate. However, the main objective of this study was to mutate the *AvrLm7* gene in the isolate that interacts with the *Rlm7* gene from *B. napus*. Therefore, this isolate can be adopted and used to broaden genetic resistance resources from *B. napus* genotypes.

### Production of a Mutant Isolate Using the CRISPR/Cas9 System

A total of 100 mutant isolates were picked from the growth medium after *Agrobacterium* transformation. Through PCR assays, no amplification of *AvrLm1*, *2*, *3*, 11 was observed in the collected mutant. As expected, positive PCR products could be obtained from the genomic DNA of all the mutants, with primers specific to *AvrLm4-7*.

For the 100 selected isolates, the nucleotides were edited near the genomic region of the guide sgRNA targeting Cas9 in 32 mutant isolates (Supplementary Figure S2). Only six mutants featured 2–8 bp deletions and the remaining mutant isolates were found to have single nucleotide insertion/deletions or substitutions (Supplementary Figure S2). The *AvrLm4-7* gene had two exons in *L. maculans* isolates (Parlange et al., 2009); the edited regions in the first exon produced significant amino acids variations in coding regions including deletions (Supplementary Figure S3). To screen the mutants, all the transformed isolates were inoculated onto the set of *Brassica* differentials to phenotype their pathogenicity. The disease rating scored from 1 to 3 in *B. napus Rlm7* genotype caused by other 31 mutants, indicating that there was no shift from avirulence to virulence on the genotype 01-23-2-1, which carries the *Rlm7* gene that interacts with *AvrLm7* (Supplementary Table S3).

**FIGURE 2:**
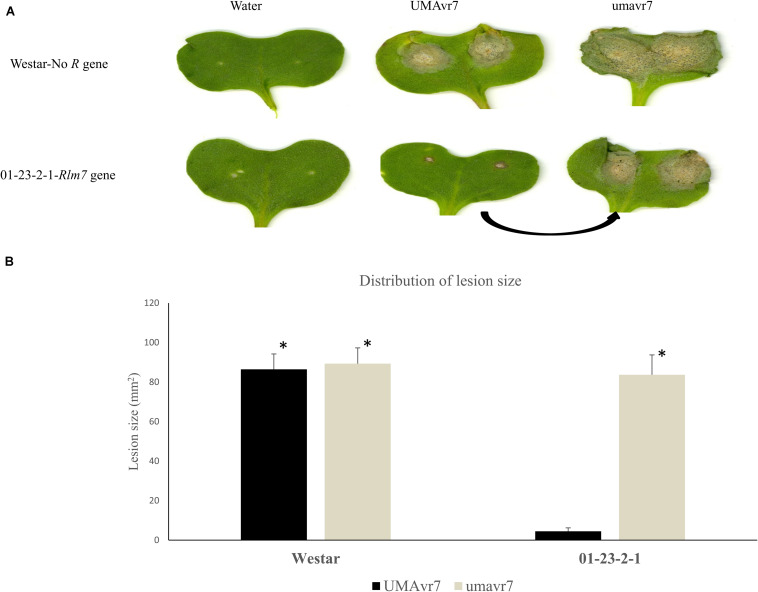
Phenotype characterization of the wild type and its mutant isolates. **(A)** Disease symptoms at 11 days post-inoculation (dpi) on Westar and 01-23-2-1 cotyledons inoculated with water, wild type isolate (UMAvr7) and the mutant isolate (umavr7). **(B)** Pathogenicity tests between wild type isolate and mutant isolate on susceptible (Westar) and resistant genotype (01-23-2-1), respectively. Each bar indicates the mean and standard deviation of lesion size for isolate on certain *Brassica napus* genotypes. The asterisk over bars represents the significant difference (*p* < 0.05) of lesion size caused by the wild type and mutant isolate.

**FIGURE 3:**
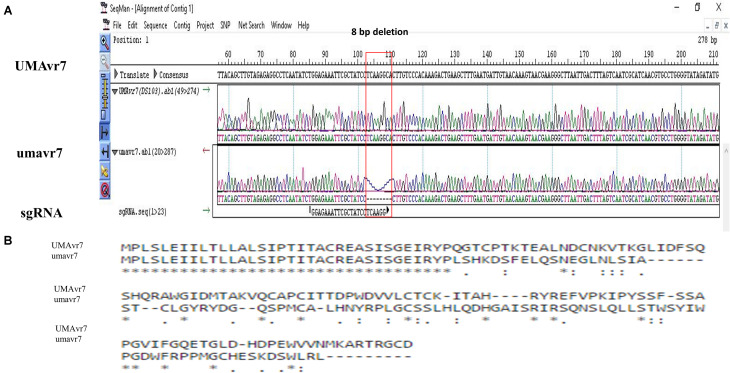
CRISPR/Cas9 mediated gene editing of *AvrLm4-7* gene of *Leptosphaeria maculans*. **(A)** The mutant isolate umavr7 carries an 8 bp deletion overlapping targeted sgRNA region. **(B)** Alignment of deduced amino acid sequences of *AvrLm4-7* with wild type isolate UMAvr7 and mutant isolate umavr7. Amino acid residues with asterisks are conserved in aligned sequences.

**FIGURE 4:**
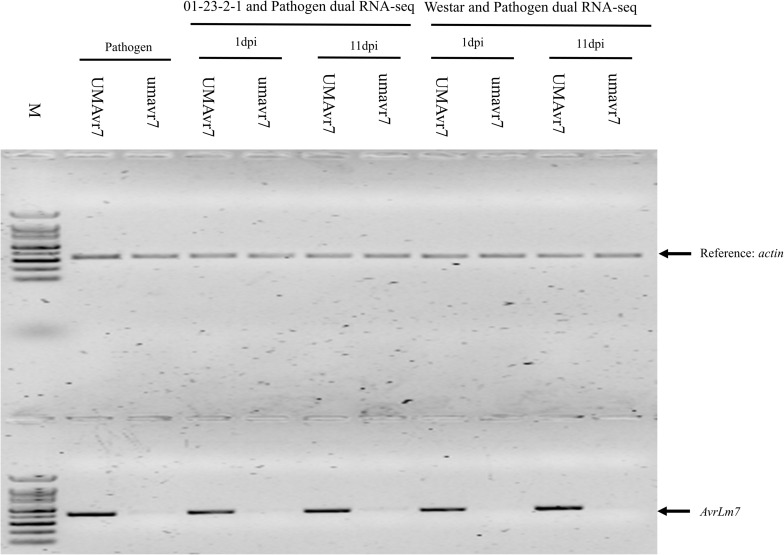
Reverse transcription (RT)-PCR analysis of *AvrLm7* gene in wild-type and mutant isolates only, and in Westar/01-23-2-1 genotypes inoculated with UMAvr7 and umavr7, respectively, at 1 dpi and 11 dpi. *Leptosphaeria maculans actin* was used as a control to quantify equal RNA using.

Among these mutants, one isolate (Mu3), hereafter termed as umavr7, when inoculated onto *B. napus* genotype 01-23-2-1 (*Rlm7*), generated large disease lesions similar to those observed in the susceptible Westar genotype (Figure 2A), indicating a shift from avirulence to virulence. The mutant isolate, umavr7, produced a significantly large lesion on 01-23-2-1, which was similar in size to lesions observed after inoculation on Westar (Figure 2B). An 8 bp deletion (TCAAGGCA) in umavr7, overlapping the sgRNA region, was confirmed by DNA sequencing. This deletion caused the amino acid change in the coding region of the *AvrLm4-7* gene in *L. maculans* UMAvr7 (Figure 3). Transcriptomes analysis in *B. napus-L. maculans* pathosystem of compatible and incompatible interaction on *Rlm7* genotype using wild type (UMAvr7) and the mutant isolate (umavr7), respectively, indicated that there is no *AvrLm7* expression in transcription data generated from umavr7 inoculation (unpublished transcriptome data). Reverse transcription (RT)-PCR assays also indicated that there is no *AvrLm7* expression in mutated isolate, and in Westar/01-23-2-1 (*Rlm7*) genotypes inoculated with mutant isolate at 1 and 11 dpi, respectively (Figure 4).

To eliminate the possibility of off-target editing by CRISPR/Cas9 system, we searched six potential off-target genomic regions from the determined genome sequence of *L. maculans*. After PCR amplification and sequencing of both wild type (UMAvr7) and mutant isolate (umavr7), there was no nucleotide polymorphism in all six potential off-target regions (Supplementary Table S4 and Supplementary Figure S4), indicating that the CRISPR/Cas9 system developed in this study specifically edited the genomic region of *AvrLm4-7* gene. Taken together, the pathogenicity of the mutant isolate umavr7 shifted from avirulent to virulent on the *B. napus* genotype harboring the *Rlm7* gene, based on the cotyledon inoculation test and PCR genotyping validation. Therefore, the mutant isolate that caused significant disease symptoms on *B. napus* genotypes with known *R* genes can be considered a virulent isolate for *B. napus* genotypes resistance screening.

### H_2_O_2_ Accumulation of Cotyledons in Response to Mutant Isolate

**FIGURE 5:**
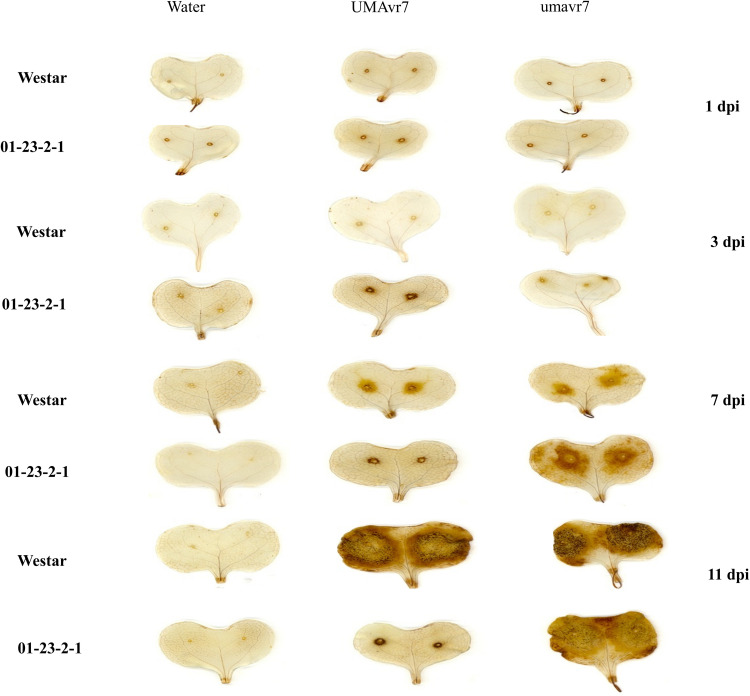
Cotyledons inhibiting H_2_O_2_ in response to *Leptosphaeria maculans* isolate UMAvr7 and umavr7 infection, and corresponding stage of cotyledons inoculated with water were set as controls. *In situ* detection of H_2_O_2_ was performed using DAB staining in the Westar and 01-23-2-1 at 1, 3, 7, and 11 days post inoculation, respectively.

*In situ* detection of H_2_O_2_ was detected in Westar and 01-23-2-1 at 1, 3, 7, and 11 dpi. Upon DAB staining, the H_2_O_2_ accumulation was not shown to be obviously different between cotyledons infected with UMAvr7 and umavr7 at an earlier stage (1–7 dpi) in both genotypes, i.e., from 1 dpi to 7 dpi. At 11 dpi, the cotyledons inoculated with UMAvr7 and umavr7 displayed a stronger reddish coloration around decayed tissue on Westar. However, in cotyledons of resistant genotype 01-23-2-1, UMAvr7 just caused limited accumulation of hydrogen peroxide around the wound site compared to mutant isolate umavr7 (Figure 5). These results demonstrated that hydrogen peroxide in cotyledons accumulate differently in response to infection with UMAvr7 and umavr7. For example, in resistant genotype 01-23-2-1, the hydrogen peroxide accumulation increased when infected with the mutant isolate at a late stage of infection.

### *B. napus* Germplasms Resistance Screening

Based on disease rating scores averaged from at least six plants with 24 wounded lobes, 115 *B. napus* genotypes displayed susceptible disease symptoms (6.1–9.0), meaning the mutant isolate was virulent on most of the genotypes carrying *Rlm1*, *Rlm2*, *Rlm3*, and *Rlm4*, or those without any of the known *R* genes (Supplementary Table S5). Two *B. napus* genotypes, 1055 and 8013, carrying *Rlm2*/unknown and *Rlm3*, respectively, showed intermediate resistance to the mutant isolate (umavr7). Interestingly, six genotypes, including 1056, 8011, 8037, HP49, CC01, and HP15, showed clear resistance to the mutant isolate (umavr7). Among the six genotypes, 8011, HP49 and HP15 were found to carry an unknown resistant gene, when characterized in a previous study (Zhang et al., 2017). The other three germplasm, 1056, 8037, and CC01, were found to have *Rlm2*/unknown, *Rlm2*/*Rlm3*/unknown, and *Rlm3*/unknown genes, respectively (Zhang et al., 2017). Theoretically, any *B. napus* genotype showing resistance to the mutant isolate has a high possibility of harboring a novel resistant gene, as the isolate lacks all of the identified avirulence genes that interact with resistance genes from *B. napus*. Our results indicate that the mutant umavr7 isolate, which has avirulence gene (*AvrLm7*) knocked out, can be used to rapidly screen the unknown resistance gene in *B. napus* instead of using a large number of differential *L. maculans* isolates.

## Discussion

This paper reports the generation of an avirulence gene (*AvrLm7*) mutation in a *L. maculans* strain, using the gene-editing tool CRISPR/Cas9. The mutant isolate caused significant disease symptoms in all the *B. napus* genotypes carrying known *R* genes, especially in 01-23-2-1, in which disease pathogenicity changed from avirulent in the wild-type isolate to virulent in the mutant. We applied the mutant umavr7 isolate to screening novel *R* genes in *B. napus* genotypes.

Currently, breeding programs aimed at developing resistance to blackleg rely on the gene-for-gene interaction model between *Brassica* species and *L. maculans*, with the latter carrying the avirulence gene that selects the corresponding *R* gene in the former. To date, a total of 19 major resistance genes have been identified and reported in several *Brassica* species, providing information on race-specificity of *L. maculans* resistance. In these studies, *L. maculans* isolates carrying different *Avr* genes were inoculated onto plants to phenotype disease severity, including “P042” (*AvrLm1*, *AvrLmS*) (Larkan et al., 2013) and “165” (*avrLm1*, *AvrLm2*, *avrLm3*) (Larkan et al., 2015), which were adopted for *Rlm1* and *Rlm2* gene identification. Two isolates harboring different *Avr* gene race structures were inoculated on *Brassica* crops, to evaluate blackleg disease resistance and identify corresponding resistance genes. Because the isolates used carried a different number of *Avr* genes, their pathogenicity on different canola genotypes varied from virulence to avirulence. Therefore, broadening the genetic bases of resistance through interspecific or intergeneric hybridization to improve resilience against blackleg disease is an important strategy in canola plant breeding. To address this issue, generating a virulent strain of *L. maculans*, which can cause blackleg disease in *B. napus* (including those harboring a specific *R* gene), is essential for screening resistant phenotypes in a breeding program. In a disease survey conducted in Western Canada, 180 *Avr* gene races were identified, with several major races, including *AvrLm2-4-5-6-7-11*, *AvrLm2-4-5-6-7-11-S*, and *AvrLm1-4-5-6-7-11-(S)*, accounting for approximately 25% of the population (Fernando et al., 2018). However, these isolates can only be used to screen the comparatively lower level resistant *Brassica* genotypes that show specific resistance to the designated *L. maculans* isolates with corresponding *Avr* genes. In contrast, the mutant isolate umavr7, generated via a genome editing tool, theoretically carries none of the known *Avr* genes and can cause all *B. napus* genotypes (with *R* gene resistance) to exhibit disease symptoms. Therefore, this mutant isolate has the potential for a wide range of uses in the selection of *B. napus* genotype with strong resistance to blackleg disease.

*B. napus* (AACC, 2*n* = 38) is a relatively young species derived from spontaneous interspecific hybridization between *Brassica rapa* (AA, 2*n* = 20) and *Brassica oleracea* (CC, 2*n* = 18). In addition, embryo rescue has been applied successfully to overcome the barrier of interspecies self-incompatibility and to increase the rate of crossing (Tonguc and Griffiths, 2004), by manipulation of the extremely high level of resistance exhibited by *B. napus* and resynthesized lines to mutant strains of *L. maculans* through breeding. In our investigation, the mutant isolate umavr7 was further evaluated on 123 *B. napus* genotypes, only six of which showed blackleg disease resistance. As is our understanding, these six *B. napus* genotypes should harbor novel *R* gene(s). This result is consistent with that previously reported by Zhang et al. (2017), who found 12 accessions exhibiting unknown *R* genes in greenhouse and field trials. However, in comparison to the identification of *B. napus* seedling or adult plant germplasm resistance to blackleg disease, using 30 different *L. maculans* isolates, carried out by Zhang et al. (2017), the application of the mutated isolate umavr7 enables an easier and more rapid route to identify resistant genotypes, as well as the identification of genotypes carrying novel/unknown *R* gene(s). These resistant genotypes can then be used for blackleg disease breeding and novel *R* gene identification. In the case of the 19 identified *R* genes, most have been mapped to the “A” genome of *B. napus* or *B. rapa* (*Rlm1-4*, *Rlm7* and *LepR1-4*) via linkage mapping. Introducing the genes conferring resistance to *L. maculans* into *B. rapa* (A genome), and *B. oleracea* (C genome) is another option for enlarging the gene pool for canola disease breeding via interspecific crossing. Individuals of *B. rapa* or *B. oleracea* accessions, screened by mutant isolates, can then be crossed and resynthesized into amphidiploid *B. napus* plants with extremely high resistance to blackleg disease. The main objective of this study was to obtain a virulent isolate that can cause disease on the *B. napus* germplasm carrying resistance genes. Then, the isolate can be adopted for breeding resistance screening.

Previously, H_2_O_2_ accumulation during *L. maculans* infection has been reported by Nováková et al. (2016). They found that the isolate having *AvrLm4-7* in the interaction led to less accumulation of hydrogen peroxide at later stages [8 and 10 days after inoculation (dai)]. We found similar results in that the wild type isolate UMAvr7 carrying *AvrLm7* caused a decreased accumulation of H_2_O_2_ in incompatible interaction of cotyledons in *Rlm7* genotype, while the mutant isolate (avrLm7) with knocked out *AvrLm7* gene showed increased accumulation at 7 and 11 dpi. This increased accumulation of H_2_O_2_ was similar to the compatible reactions on the susceptible genotype “Westar” caused by both wild type and mutant isolates. This demonstrates that H_2_O_2_ plays important roles in the defense response to *L. maculans* infection. Nováková et al. (2016) also indicated that *rbohF* encoding one of the NADPH oxidases for ROS (reaction oxygen species) production in the apoplast showed decreased transcription in cotyledons infected with the isolate carrying with *AvrLm4-7*. One effector gene *pep1* from the biotrophic pathogen *Ustilago maydis* has been reported to inhibit the maize peroxidase POX12 in the apoplast (Hemetsberger et al., 2012). However, we cannot prove that the *AvrLm7* will affect the accumulation of H_2_O_2_ similar to *pep1*-regulation manner without investigation of purified AvrLm7 protein effects on the ROS production; or the SA (salicylic acid) and ET (ethylene) signaling affected by ROS production or H_2_O_2_ accumulation will enhance the hypersensitive reaction (HR) to establish the defense when the plant infected with isolates having effector genes (Draper, 1997; de Jong et al., 2002). Therefore, the hypothesis of that the presence of *AvrLm7/AvrLm4-7* will reduce the accumulation of ROS and drive the HR in the *B. napus-L. maculans* pathosystem through affecting the SA and ET signaling pathways needs to be further studied. However, our observation in this study provides insights in that the isolates with the same genetic background will cause different accumulation levels of H_2_O_2_ by knocking out the effector gene *AvrLm7* and therefore, show avirulence and virulence on the *B. napus* genotype with corresponding resistance gene (*Rlm7*). Traditional approaches used to determine the functions of uncharacterized or characterized fungal genes are typically based on the identification of phenotypic expression, or the homologous recombination-mediated switching on or off of a particular gene. However, the latter method of gene targeting is not particularly efficient in fungi and is limited by incompatible mutagenesis screening on a genome-wide scale. More recently, the CRISPR/Cas9 system has been widely adopted to disrupt target genes in fungi and bacteria (Takayuki et al., 2015; Deng et al., 2017; Idnurm et al., 2017; Schuster et al., 2018).

This method represents a very important technological advance in the simultaneous disruption of functional genes/gene families to investigate their specific contribution to pathogen virulence. The *L. maculans* isolate UMAvr7, used in the present study, carried the *AvrLm7* gene, which confers a high level of virulence to canola genotypes, with the exception of those genotypes carrying the *Rlm7* gene. To obtain a mutant isolate that can be used in material screening and breeding, the *AvrLm7* gene must be mutated and its pathogenicity altered. Here we adapted CRISPR/Cas9 to disrupt a specific gene (*AvrLm7*) in the UMAvr7 isolate without the need for resistance markers. The efficiency of this CRISPR/Cas9-mediated *AvrLm7* gene editing was quite high in progeny derived from a single transformant, as demonstrated by sequencing and phenotyping, with 32% mutant progeny produced from transformation of a single isolate and with different sequence variations in the selected candidate isolates. For the editing of specific genes, CRISPR/Cas9 works easily and quickly to target the gene used to produce mutants for subsequent function analysis. Another advantage of the CRISPR/Cas9 technique is that it offers the possibility of disrupting several *Avr* genes simultaneously, by using multiple sgRNAs or a single sgRNA molecule targeting a region that is highly conserved across members of a gene family. A future interest for our research group is to use the CRISPR/Cas9 molecular tool to investigate the difference in virulence provided by a single *Avr* gene mutation and by multiple *Avr* gene mutations. In addition, the genomic and transcriptional profiling of wild-type and mutant isolates, as well as plant defense responses to infection, should provide a deeper understanding of the gene interactions between canola and the *L. maculans* pathogen.

## Data Availability Statement

All datasets presented in this study are included in the article/Supplementary Material.

## Author Contributions

ZZ and WF designed the experiments and wrote and revised the manuscript. ZZ, FL, and CS performed the experiments. ZZ, FL, and WF analyzed and interpreted the data. All authors contributed to the article and approved the submitted version.

## Conflict of Interest

The authors declare that the research was conducted in the absence of any commercial or financial relationships that could be construed as a potential conflict of interest.

## Footnotes

1 http://tools.flycrispr.molbio.wisc.edu/targetFinder/

2 https://www.dnastar.com/software/molecular-biology/

3 https://genome.jgi.doe.gov/pages/blast-query.jsf?db=Lepmu1
