# Supplementary material for: Generation and Characterization of a Virulent Leptosphaeria maculans Isolate Carrying a Mutated AvrLm7 Gene Using the CRISPR/Cas9 System
Source: Front Microbiol. 2020 Aug 11;11:1969. doi: 10.3389/fmicb.2020.01969 (PMC7432424; doi:10.3389/fmicb.2020.01969)
Supplement: Supplementary file 1 [file Table_1.docx]

### *Supplementary materials*

**Generation and characterisation of a virulent *Leptosphaeria maculans* isolate carrying a mutated *AvrLm7* gene using the CRISPR/Cas9 system**

Zhongwei Zou, Fei Liu, Carrie Selin, W. G. Dilantha Fernando*

Department of Plant Science, University of Manitoba, 66 Dafoe Road, Winnipeg, MB, R3T 2N2, Canada,

*Correspondence: W. G. Dilantha Fernando, E-mail: Dilantha.Fernando@umanitoba.ca

***Supplementary Tables***

**Supplementary Table 1** List of primer pairs for Avirulence genes amplification, CRISPR/Cas9 expression system, mutant sequencing, off target amplification and sequencing

| Primer name | sequence (5'-3') | Product size (bp) | Annotation | Reference |
| --- | --- | --- | --- | --- |
| AvrLm1-F | CTATTTAGGCTAAGCGTATTCATAAG | 1,123 | Avr gene identification | Gout et al. 2006 |
| AvrLm1-R | GCGCTGTAGGCTTCATTGTAC |  |  |  |
| AvrLm2-F | CGTCATCAATGCGTTCGG | 258 | Avr gene identification | Ghanbarnia et al. 2015 |
| AvrLm2-R | CTGGATCGTTTGCATGGA |  |  |  |
| AvrLm3-F | GAGAGAACTAGTCTGTTAAATGCCTGCTGT | 1,357 | Avr gene identification | Plissonneau et al. 2016 |
| AvrLm3-R | GAGAGACTCGAGCGCGCTTATGTTAGAATC |  |  |  |
| AvrLm4-7-F | TATCGCATACCAAACATTAGGC | 1,433 | Avr gene identification | Parlange et al. 2009 |
| AvrLm4-7-R | GATGGATCAACCGCTAACAA |  |  |  |
| AvrLmJ1/5-F | ACAACCACTCTTCTTCACAGT | 479 | Avr gene identification | Van de Wouw et al. 2013 |
| AvrLmJ1/5-R | TGGTTTGGGTAAAGTTGTCCT |  |  |  |
| AvrLm6-F | TCAATTTGTCTGTTCAAGTTATGGA | 774 | Avr gene identification | Fudal et al. 2007 |
| AvrLm6-R | CCAGTTTTGAACCGTAGTGGTAGCA |  |  |  |
| AvrLm11-F | TGCGTTTCTTGCTTCCTATATTT | 359 | Avr gene identification | Balesdent et al. 2013 |
| AvrLm11-R | CAAGTTGGATCTTTCTCATTCG |  |  |  |
| Cs-1inF | TCCGCTGAGGgtttaGGGTTTAATGCGTAAGCTCCCT | - | CRISPR/Cas9 expression | This study |
| Cs-2inR | tcggctgaggtcttaGGTCTTAATGAGCCAAGAG |  |  |  |
| Avr4-7gRNA-F | AAACGAGTAAGCTCGTCGGAGAAATTCGCTATCCTCAGTTTTAGAGCTAGAAATAGCAAG | - | CRISPR on target sgRNA | This study |
| Avr4-7gRNA-R | CGAGCTTACTCGTTTCGTCCTCACGGACTCATCAGGGAGAACGGTGATGTCTGCTCAAGC |  |  |  |
| Seq4-7 F | atgccactatccctcgagataatc | 400 bp | Mutant sequencing | This study |
| Seq4-7 R | agggaattttgggaacgaat |  |  |  |
| Lmoff-1F | TATATTTCAAGGGTGTCGACAATA | 362 bp | Off target PCR and sequencing | This study |
| Lmoff-1R | TTTCCTTTAGACTTTTGACTGAAC |  |  |  |
| Lmoff-2F | ATACGACATTCTCAAGAAAGAAGA | 341 bp | Off target PCR and sequencing | This study |
| Lmoff-2R | ATTTCTCACAGCTTTTACAATGTT |  |  |  |
| Lmoff-3F | ACGTCAGAAATTGAACTAGTAAGC | 410 bp | Off target PCR and sequencing | This study |
| Lmoff-3R | CGTTGATTTCATTCTCTTTGTAAT |  |  |  |
| Lmoff-4F | GTGTTTTGCTCGTCTGTTCT | 442 bp | Off target PCR and sequencing | This study |
| Lmoff-4R | GATTTTACAACACAGAGAGAAGGT |  |  |  |
| Lmoff-5F | GCTGAGAACCTCCTCAGCCTT | 162 bp | Off target PCR and sequencing | This study |
| Lmoff-5R | TGGGTGAAGATGCAAACAAA |  |  |  |
| Lmoff-6F | AGACATTGTCAAGGGTATCATC | 479 bp | Off target PCR and sequencing | This study |
| Lmoff-6R | TCTTCCTATCTGAATCTCTTTGTC |  |  |  |

**Supplementary Table 2.** *Brassica* genotypes used to differentiate avirulence profiles of wild type and mutant isolates.

| *Brassica* species | Cultivar/line | Host resistance genotype | Reference |
| --- | --- | --- | --- |
| *B. napus* | 01-23-2-1 | *Rlm7* | Dilmaghani *et al*. 2009 |
| *B. napus* | Quinta | *Rlm1, Rlm3* | Kutcher *et al*. 2010 |
| *B. napus* | Surpass 400 | *LepR3, RlmS* | Larkan *et al*. 2013 |
| *B. napus* | 1065 | *LepR1* | Kutcher *et al*. unpublished |
| *B. napus* | Glacier | *Rlm2 Rlm3* | - |
| *B. napus* | 1135 | *LepR2* | Kutcher *et al.* unpublished |
| *B. napus* | Jet Neuf | *Rlm4* | Gout et al. 2006 |
| *B. napus* | *Goéland* | *Rlm9* | Balesdent *et al*. 2006 |
| *B. napus* | 02-22-2-1 | *Rlm3* | Gout *et al*. 2006 |
| *B. napus* | Westar | No resistance gene | Balesdent *et al*. 2002 |
| *B. juncea* | Forge | *Rlm6* | - |

**Supplementary Table 3**: Mean disease rating scores and their inferred phenotypes for seedlings of *Brassica napus* genotypes Westar (No *R* gene) and 01-23-2-1 (*Rlm7*) in response to wild type *Leptosphaeria maculas* isolate UMAvr7 and its mutants.

|  | Disease Rating | | |
| --- | --- | --- | --- |
| Isolate ID | Westar/No *R* | | 01-23-2-1/*Rlm7* |
| UMAvr7^a^ | | 9.00 (S)^c^ | 1.87 (R) |
| Mu1 | | 8.33 (S) | 2.50 (R) |
| Mu2 | | 9.00 (S) | 3.00 (R) |
| Mu3 (umavr7)^b^ | | 9.00 (S) | 8.33 (S) |
| Mu4 | | 8.33 (S) | 2.33 (R) |
| Mu5 | | 7.87 (S) | 1.87 (R) |
| Mu6 | | 8.00 (S) | 3.00 (R) |
| Mu7 | | 7.50 (S) | 2.50 (R) |
| Mu8 | | 8.33 (S) | 1.33 (R) |
| Mu9 | | 8.33 (S) | 1.67 (R ) |
| Mu10 | | 7.87 (S) | 2.33 (R) |
| Mu11 | | 7.50 (S) | 2.13 (R) |
| Mu12 | | 9.00 (S) | 3.00 (R) |
| Mu13 | | 8.33 (S) | 2.50 (R) |
| Mu14 | | 7.87 (S) | 1.67 (R) |
| Mu15 | | 8.00 (S) | 2.13 (R) |
| Mu16 | | 7.50 (S) | 3.00 (R) |
| Mu17 | | 7.25 (S) | 1.67 (R) |
| Mu18 | | 8.33 (S) | 1.33 (R) |
| Mu19 | | 7.00 (S) | 3.00 (R) |
| Mu20 | | 7.00 (S) | 1.87 (R) |
| Mu21 | | 8.00 (S) | 3.00 (R) |
| Mu22 | | 8.33 (S) | 2.50 (R) |
| Mu23 | | 7.50 (S) | 1.67 (R) |
| Mu24 | | 8.15 (S) | 2.13 (R) |
| Mu25 | | 8.33 (S) | 3.00 (R) |
| Mu26 | | 8.50 (S) | 3.00 (R) |
| Mu27 | | 8.00 (S) | 1.87 (R) |
| Mu28 | | 7.87 (S) | 3.00 (R) |
| Mu29 | | 8.33 (S) | 2.33 (R) |
| Mu30 | | 7.50 (S) | 2.13 (R) |
| Mu31 | | 8.33 (S) | 3.00 (R) |
| Mu32 | | 7.87 (S) | 3.00 (R) |

^a^ Wild type isolate used in this study (DS103).

^b^ Mutant isolate umavr7 altering pathogenicity from avriulent to virulent on *Rlm7* genotype.

^c^ R, IR, S = *Brassica napus* genotypes that displayed resistance, intermediate resistance, and susceptibility, respectively.

**Supplementary Table 4.** Potential CRISPR/Cas9 off targets validation from *Leptospheria maculans* genome.

| **Overlapping gene** | **Overlapped length sgRNA** | **Overlapped sgRNA sequence** | **PCR included region size** | **Polymorphism (UMAvr7 vs umavr7)** |
| --- | --- | --- | --- | --- |
| LMEA P070090.1 | 15 bp | TCGCTATCCTCAAGG | 362 bp | NO |
| LMEA P103860.1 | 14 bp | GGAGAAATTCGCTA | 341 bp | NO |
| LMEA uP059110.1 | 13 bp | AAATTCGCTATCC | 410 bp | NO |
| LMEA P044630.1 | 13 bp | TTCGCTATCCTCA | 442 bp | NO |
| LMEA P014240.1 | 13 bp | TTCGCTATCCTCA | 162 bp | NO |
| LMEA P010470.1 | 13 bp | TTCGCTATCCTCA | 479 bp | NO |

| **Supplementary Table 5**. Pathogenicity test on 123 *Brassica napus* genotypes using mutant isolate umavr7 | | | |
| --- | --- | --- | --- |
| *Brassica napus* ID^a^ | Resistance genoype^b^ | Disease score^c^ | Inferred phenotype^d^ |
| 1017 | *Rlm3* | 6.75 | *S* |
| 1021 | *Rlm3* | 7.25 | *S* |
| 1037 | *Rlm3* | 7.33 | *S* |
| 1051 | None | 6.67 | *S* |
| 1055 | *Rlm2,* unknown | 5.25 | *IR* |
| 1056 | *Rlm2,* unknown | 1.33 | *R* |
| 1058 | *Rlm3* | 6.15 | *S* |
| 1068 | *Rlm3* | 6.25 | *S* |
| 1075 | none | 7.33 | *S* |
| 8011 | Unknown | 1.50 | *R* |
| 8012 | None | 7.00 | *S* |
| 8013 | None | 7.33 | *S* |
| 8014 | *Rlm3* | 5.25 | *IR* |
| 8015 | *Rlm4* | 7.00 | *S* |
| 8016 | *Rlm4* | 9.00 | *S* |
| 8017 | None | 7.50 | *S* |
| 8021 | *Rlm3* | 6.83 | *S* |
| 8022 | None | 7.50 | *S* |
| 8024 | None | 9.00 | *S* |
| 8025 | None | 7.50 | *S* |
| 8026 | None | 8.33 | *S* |
| 8027 | *Rlm3* | 8.00 | *S* |
| 8030 | None | 7.50 | *S* |
| 8034 | Unknown | 8.00 | *S* |
| 8037 | *Rlm2,Rlm3,*unknown | 1.50 | *R* |
| 8041 | *Rlm2,* unknown | 7.33 | *S* |
| 8048 | None | 8.00 | *S* |
| HC1022 | *Rlm4* | 7.00 | *S* |
| HC1023 | None | 9.00 | *S* |
| HC1024 | None | 8.33 | *S* |
| HC1025 | None | 7.50 | *S* |
| HC1026 | None | 8.33 | *S* |
| HC1027 | *Rlm3* | 6.83 | *S* |
| HC1028 | *Rlm4* | 7.55 | *S* |
| HC701 | None | 9.00 | *S* |
| HC702 | None | 9.00 | *S* |
| HC703 | None | 8.33 | *S* |
| HC801 | *Rlm3* | 7.50 | *S* |
| HC802 | None | 7.50 | *S* |
| HC803 | None | 6.83 | *S* |
| HC804 | *Rlm3* | 7.33 | *S* |
| HC805 | None | 9.00 | *S* |
| HC806 | None | 7.50 | *S* |
| HC807 | *Rlm3* | 8.33 | *S* |
| HC808 | None | 9.00 | *S* |
| HC809 | None | 7.33 | *S* |
| HC811 | None | 6.83 | *S* |
| HC812 | None | 7.00 | *S* |
| HC813 | *Rlm3* | 9.00 | *S* |
| HC814 | None | 8.33 | *S* |
| HC815 | None | 9.00 | *S* |
| HP26 | *Rlm4* | 6.83 | *S* |
| HP27 | *Rlm4* | 7.00 | *S* |
| HP28 | *Rlm1* | 8.00 | *S* |
| HP29 | None | 8.33 | *S* |
| HP30 | None | 9.00 | *S* |
| HP31 | None | 6.83 | *S* |
| HP32 | None | 7.00 | *S* |
| HP33 | *Rlm3* | 8.33 | *S* |
| HP34 | None | 9.00 | *S* |
| HP35 | None | 6.83 | *S* |
| HP36 | *Rlm4* | 7.50 | *S* |
| HP37 | *Rlm1* | 6.67 | *S* |
| HP38 | None | 7.33 | *S* |
| HP39 | None | 7.00 | *S* |
| HP40 | None | 9.00 | *S* |
| HP41 | None | 8.33 | *S* |
| HP42 | None | 7.50 | *S* |
| HP43 | *Rlm4* | 7.00 | *S* |
| HP44 | None | 9.00 | *S* |
| HP45 | None | 7.00 | *S* |
| HP46 | *Rlm4* | 8.33 | *S* |
| HP47 | None | 7.00 | *S* |
| HP48 | None | 8.33 | *S* |
| HP49 | Unknown | 1.50 | *R* |
| CC01 | *Rlm3,* unknown | 1.67 | *R* |
| CC02 | *Rlm3* | 6.85 | *S* |
| CC03 | *Rlm3* | 8.33 | *S* |
| CC04 | *Rlm3* | 7.00 | *S* |
| CC05 | None | 9.00 | *S* |
| CC06 | None | 6.85 | *S* |
| CC07 | None | 7.00 | *S* |
| CC08 | None | 9.00 | *S* |
| CC09 | None | 8.33 | *S* |
| CC10 | *Rlm4* | 7.50 | *S* |
| HC1001 | None | 9.00 | *S* |
| HC1002 | None | 7.00 | *S* |
| HC1003 | None | 6.85 | *S* |
| HC1004 | *Rlm3* | 7.00 | *S* |
| HC1005 | None | 8.33 | *S* |
| HC1007 | None | 9.00 | *S* |
| HC1008 | None | 6.85 | *S* |
| HC1010 | None | 7.50 | *S* |
| HC1011 | None | 7.33 | *S* |
| HC1012 | None | 8.33 | *S* |
| HC1013 | None | 7.00 | *S* |
| HC1014 | *Rlm3* | 6.85 | *S* |
| HC1015 | None | 7.00 | *S* |
| HC1016 | None | 9.00 | *S* |
| HC1017 | None | 8.33 | *S* |
| HC1018 | None | 7.50 | *S* |
| HC1019 | None | 6.85 | *S* |
| HC1020 | None | 7.00 | *S* |
| HP01 | *Rlm3* | 7.00 | *S* |
| HP02 | None | 9.00 | *S* |
| HP03 | None | 8.33 | *S* |
| HP04 | None | 6.85 | *S* |
| HP05 | None | 8.33 | *S* |
| HP06 | None | 9.00 | *S* |
| HP08 | *Rlm4* | 7.50 | *S* |
| HP09 | *Rlm1* | 8.33 | *S* |
| HP10 | *Rlm3* | 6.25 | *S* |
| HP11 | None | 7.00 | *S* |
| HP12 | None | 6.85 | *S* |
| HP13 | None | 8.33 | *S* |
| HP14 | None | 7.00 | *S* |
| HP15 | Unknown | 2.33 | *R* |
| HP16 | None | 7.00 | *S* |
| HP17 | *Rlm4* | 8.33 | *S* |
| HP20 | *Rlm4* | 9.00 | *S* |
| HP22 | *Rlm4* | 7.33 | *S* |
| HP23 | *Rlm4* | 7.00 | *S* |
| HP24 | Unknown | 6.85 | *S* |

^a^ A collection of Chinese *Brassica napus* genotypes including 123 varieties or advanced breeding lines (Zhang et al. 2017).

^b^ Characterized resistance genes (Zhang et al. 2017).

^c^ Disease score averaged from at least six plants.

^d^ R, IR, S = *Brassica napus* genotypes that displayed resistance, intermediate resistance, and susceptibility, respectively, to mutant isolate umavr7*.*

***Supplementary Figures***

Supplementary Figure 1


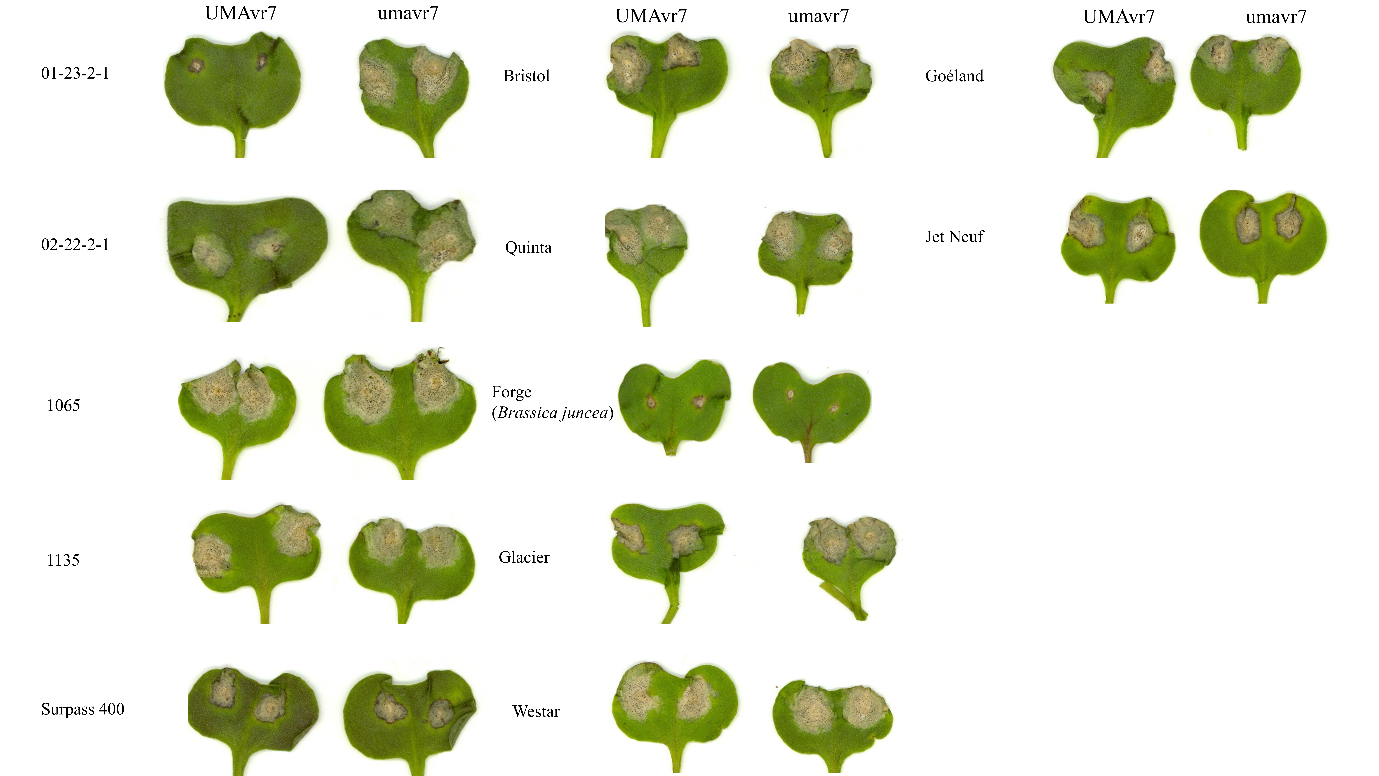


**Supplementary Figure 1.** Phenotypic characterisation of the wild type and its mutant isolates on 12 *B. napus* differentials. Cotyledons showing disease symptoms were scanned at 14 days post-inoculation (dpi).

Supplementary Figure 2


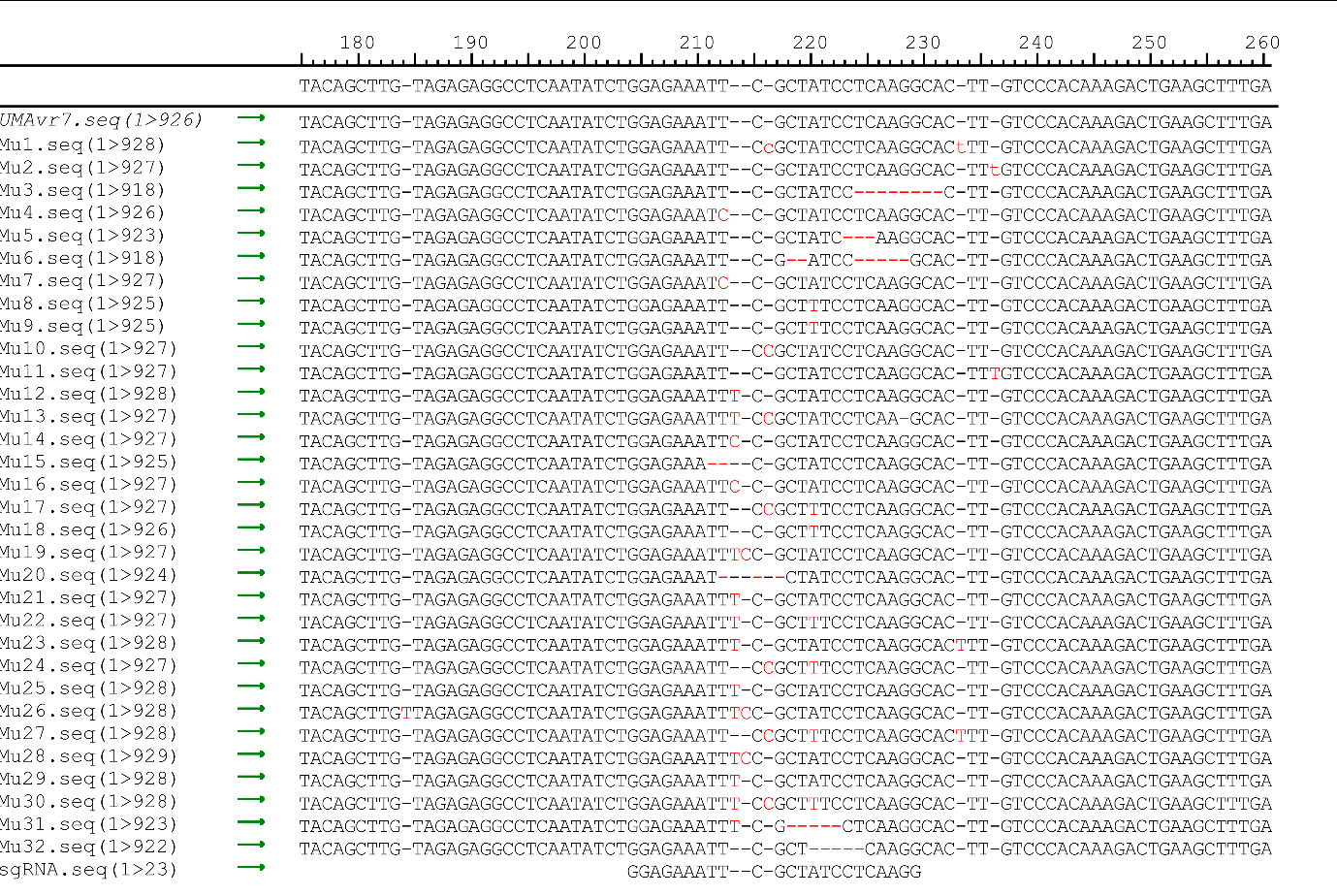


**Supplementary Figure 2.** Alignment of *AvrLm4-7* gene sequences from wild type isolate (UMAvr7) and 32 CRISPR/Cas9 generated mutantsGuide sgRNA sequence from *AvrLm4-7* gene is listed at the bottom of alignment. Mu3 was the mutant isolate selected in this study. All the mutations detected in mutants are highlighted in red**.**

Supplementary Figure 3


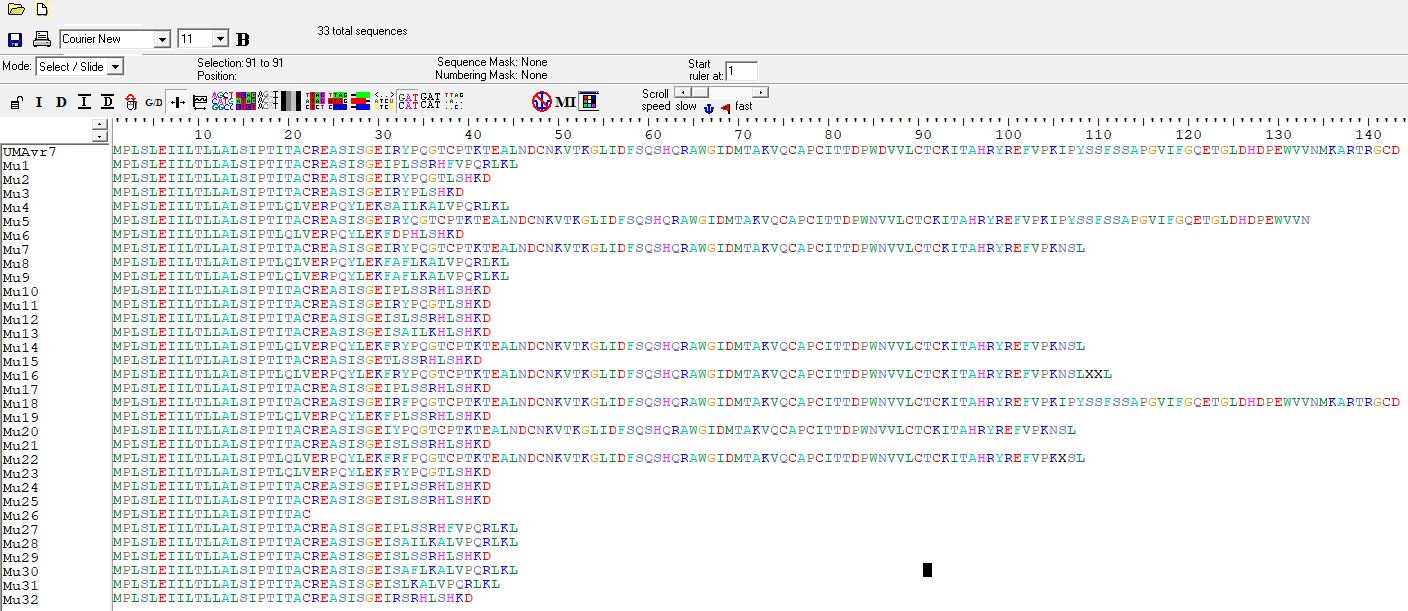


**Supplementary Figure 3.** Alignment of deduced amino acids sequences of wild-type isolate (UMAvr7) and 32 CRISPR/Cas9 generated mutants. The sequence alignment was conducted in BioEdit (http://www.mbio.ncsu.edu/BioEdit/bioedit.html).

Supplementary Figure 4


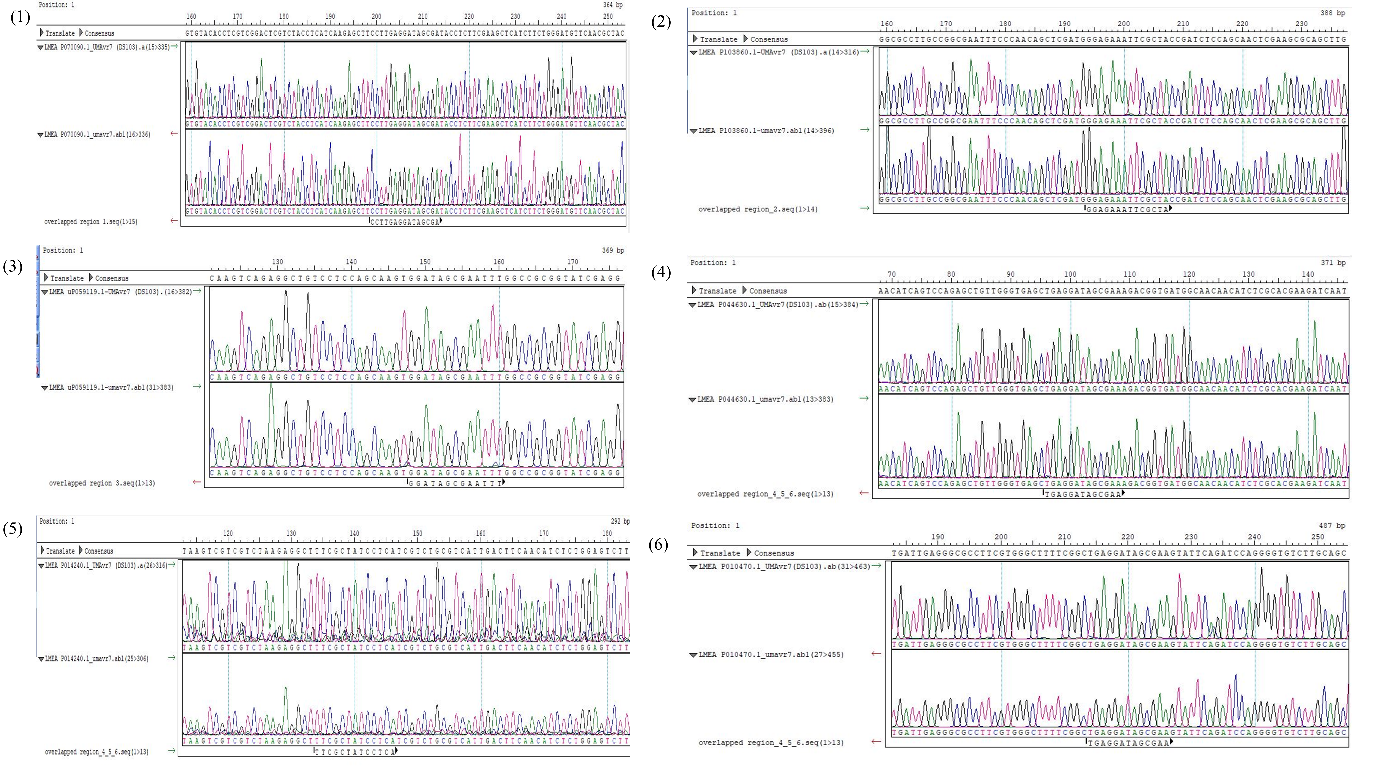


**Supplementary Figure 4.** Sequence alignments of six CRISPR/Cas9 off-target regions (1-6) between wild type isolate (UMAvr7) and mutant isolate (umavr7).
